# Risk factors, subtype profiles, and outcomes of cardiac rupture after acute myocardial infarction: a case-control study

**DOI:** 10.3389/fcvm.2026.1827832

**Published:** 2026-06-10

**Authors:** Dan Zhang, Chaojie Huang, Xiaosu Wang, Jingdan Yu, Litao Zhang, Bo Liu

**Affiliations:** 1 Department of Clinical Laboratory, Wuhan Asia General Hospital, Wuhan Asia General Hospital Affiliated to Wuhan University of Science and Technology, Wuhan, China; 2 Department of Critical Care Medicine, Wuhan Asia General Hospital, Wuhan Asia General Hospital Affiliated to Wuhan University of Science and Technology, Wuhan, China

**Keywords:** acute myocardial infarction, cardiac rupture, case-control study, free wall rupture, risk factors, ventricular septal rupture

## Abstract

**Background:**

Cardiac rupture (CR) is a fatal mechanical complication of acute myocardial infarction (AMI) with mortality rates exceeding 50%. Early identification of high-risk patients remains clinically important, especially in regions with delayed presentation and limited contemporary data.

**Methods:**

This retrospective case-control study included 71 unique patients with CR [free wall rupture (FWR) 39, ventricular septal rupture (VSR) 28, papillary muscle rupture (PMR) 4] and 213 AMI controls without mechanical complications (1:3 ratio, stratified by AMI type) from a tertiary hospital in Wuhan, China (June 2020–December 2025). Factors independently associated with CR were identified using multivariable logistic regression with the events-per-variable principle (≥10). Model discrimination was assessed by the C-statistic and calibration by the Hosmer-Lemeshow test. Subtype comparisons (FWR vs. VSR), early (≤3 days) vs. late (>3 days) rupture analysis, and in-hospital mortality correlates were also evaluated.

**Results:**

Patients with CR were older (69.6 ± 8.5 vs. 59.5 ± 11.7 years, *P* < 0.001), more frequently female (40.8% vs. 12.2%, *P* < 0.001), and had longer onset-to-door times (median 48 vs. 5 h, *P* < 0.001). In multivariable analysis, absence of emergency percutaneous coronary intervention (PCI) [odds ratio (OR) = 8.23, 95% confidence interval (CI) 3.45–19.66], Killip class III–IV (OR = 6.82, 95% CI 2.68–17.35), female sex (OR = 3.41, 95% CI 1.41–8.26), and lower serum albumin (OR = 0.84 per g/L, 95% CI 0.77–0.91) were independently associated with CR. FWR showed markedly higher mortality than VSR (97.4% vs. 57.1%, *P* < 0.001). Early rupture (≤3 days, 39.4%) was associated with more intense inflammatory activation and higher admission troponin, whereas late rupture (>3 days, 60.6%) was associated with lower albumin and markedly delayed presentation, appearing to correspond to different temporal clinical patterns. Surgical repair was the only factor associated with lower in-hospital mortality in the multivariable model (OR = 0.06, 95% CI 0.01–0.36, *P* = 0.002).

**Conclusions:**

Absence of emergency PCI, Killip class III–IV, female sex, and hypoalbuminemia were independently associated with CR after AMI. FWR and VSR showed different clinical and hemodynamic patterns. Surgical repair was associated with lower in-hospital mortality among selected CR patients, supporting prompt multidisciplinary evaluation while recognizing the likelihood of survivor and indication bias.

## 1 Introduction

Cardiac rupture (CR) is one of the most devastating mechanical complications following acute myocardial infarction (AMI), encompassing free wall rupture (FWR), ventricular septal rupture (VSR), and papillary muscle rupture (PMR). Although the widespread adoption of primary percutaneous coronary intervention (PCI) has reduced its incidence substantially, CR remains a leading cause of in-hospital death after AMI, with mortality rates ranging from 50% to over 90% depending on the subtype (1–3). The abrupt onset and rapidly fatal course of CR, particularly FWR, leave an extremely narrow window for therapeutic intervention, making early identification of high-risk patients a clinical priority.

Multiple risk factors for CR have been identified in prior studies, including advanced age, female sex, first myocardial infarction, anterior infarct location, higher Killip class, and absence of reperfusion therapy (2–4). A recent systematic review and meta-analysis of ten prediction models reported a pooled C-statistic of 0.83 for CR prediction, with age, female sex, Killip grade, and PCI status identified as the most consistent predictors (5). However, the existing literature has several notable gaps. First, the majority of studies originate from East Asian centers in eastern and northern China, Japan, and Korea, or from European and North American registries; data from central China remain scarce. Second, most prior reports have focused on a single subtype of CR (predominantly FWR) or analyzed all subtypes as a composite outcome, with few studies directly comparing the clinical profiles of FWR and VSR within the same cohort. Third, the temporal pattern of CR and its relationship to clinical characteristics have not been fully characterized.

Given the high case-fatality rate and the potential clinical value of earlier recognition, there is a clear need for studies that not only validate established risk factors in underrepresented populations but also provide detailed comparisons across CR subtypes and temporal patterns, areas that remain insufficiently characterized in the existing literature.

Therefore, the present study aimed to: (1) identify factors independently associated with CR after AMI in a single tertiary center in central China; (2) compare the clinical characteristics and outcomes of FWR and VSR; and (3) examine the temporal distribution and in-hospital mortality correlates of CR.

## 2 Methods

### 2.1 Study design and population

This was a retrospective, single-center, case-control study conducted at a tertiary hospital in Wuhan, central China. Consecutive patients diagnosed with AMI who were admitted between June 2020 and December 2025 were screened for eligibility. AMI was diagnosed according to the Fourth Universal Definition of Myocardial Infarction (6). The study was approved by the institutional ethics committee, and the requirement for individual informed consent was waived given the retrospective nature of the study. All data were de-identified prior to analysis.

The case group comprised patients who developed CR during hospitalization, confirmed by echocardiography, surgical findings, or autopsy. CR was classified into three mutually exclusive subtypes: FWR, VSR, and PMR. Each patient was counted once. The control group consisted of AMI patients without mechanical complications during the same hospitalization period, randomly selected at a 1:3 case-to-control ratio using computer-generated random sampling stratified by AMI type (STEMI vs. NSTEMI) and year of admission to ensure temporal and diagnostic comparability. No matching by age or sex was performed, as these were prespecified candidate predictor variables; matching on them would have precluded estimation of their independent associations with CR.

Inclusion criteria were: (1) age ≥18 years; (2) confirmed diagnosis of AMI; (3) for cases, confirmed CR during hospitalization; and (4) for controls, absence of any mechanical complications during hospitalization. Exclusion criteria were: (1) missing more than 30% of key clinical data; (2) coexisting severe structural heart disease (e.g., hypertrophic cardiomyopathy or severe valvular disease unrelated to AMI); and (3) traumatic cardiac rupture.

### 2.2 Diagnostic criteria for cardiac rupture

FWR was diagnosed based on the presence of sudden hemodynamic collapse or electromechanical dissociation, echocardiographic evidence of significant pericardial effusion, and confirmation by bloody pericardiocentesis, surgery, or autopsy. VSR was diagnosed by the detection of a new systolic murmur accompanied by echocardiographic demonstration of a left-to-right shunt at the ventricular septal level. PMR was diagnosed by the acute onset of severe mitral regurgitation with echocardiographic evidence of papillary muscle rupture or prolapse.

### 2.3 Data collection

Clinical data were retrospectively extracted from the electronic medical record system by two investigators independently, with discrepancies resolved by a third reviewer. The following variables were collected: demographic characteristics, medical history, clinical presentation, reperfusion treatment, laboratory indices, echocardiographic findings, rupture subtype, management strategies, and in-hospital outcomes. Medication variables, including dual antiplatelet therapy, beta-blockers, and ACEI/ARB, were defined according to treatment exposure before the diagnosis of cardiac rupture. IABP use was defined as pre-rupture mechanical circulatory support initiated in high-risk AMI patients before the diagnosis of cardiac rupture.

### 2.4 Study outcomes

The primary outcome was the occurrence of cardiac rupture after AMI. Secondary outcomes included in-hospital all-cause mortality within the CR group and comparison of clinical characteristics between CR subtypes (FWR vs. VSR) and between early (≤3 days) and late (>3 days) rupture.

### 2.5 Statistical analysis

Continuous variables were assessed for normality using the Shapiro–Wilk test (*n* < 50) or D'Agostino-Pearson test (*n* ≥ 50). Normally distributed continuous variables are presented as mean ± standard deviation and were compared using the independent-samples *t*-test. Non-normally distributed continuous variables are presented as median [interquartile range (IQR)] and were compared using the Mann–Whitney *U*-test. Categorical variables are expressed as counts (percentages) and were compared using the chi-square test or Fisher's exact test, as appropriate. Complete data were available for all 284 participants for the variables included in the primary multivariable model. For certain laboratory variables (e.g., NT-proBNP), a small number of values were missing; the available sample sizes are indicated in the supplementary Figures.

To identify factors independently associated with cardiac rupture, univariate logistic regression was first performed for all candidate variables. Variables with *P* < 0.1 in univariate analysis, together with age and sex, were considered for entry into the multivariable model. Variable selection further considered clinical plausibility, data completeness, and collinearity to avoid overfitting. Given the number of CR events (*n* = 71), the final full model was limited to seven variables to maintain an events-per-variable ratio of approximately 10:1. A parsimonious model retaining only the independently associated variables was then constructed. Model discrimination was assessed using the C-statistic (area under the receiver operating characteristic curve), and calibration was assessed using the Hosmer-Lemeshow goodness-of-fit test.

For the comparison between FWR and VSR subtypes, the same descriptive and comparative methods were applied. PMR cases (*n* = 4) were described separately and excluded from the subtype comparison due to the extremely small sample size. For the temporal analysis, CR cases were categorized as early rupture (≤3 days from AMI onset) or late rupture (>3 days) and compared using the same statistical methods. The 72-hour cutoff was selected *a priori* based on the recognized pathological transition from acute transmural coagulative necrosis to inflammatory infarct expansion and matrix metalloproteinase–mediated remodeling, consistent with classical pathological descriptions and contemporary clinical series of post-infarction cardiac rupture (7, 8). The robustness of the qualitative findings to this choice was examined in sensitivity analyses using alternative cutoffs of ≤2 and ≤4 days (Supplementary Table S3).

In-hospital mortality within the CR group was analyzed using logistic regression. Univariate analysis was performed to screen candidate predictors, followed by multivariable analysis for variables with *P* < 0.1 and strong clinical relevance. The final mortality model included three variables (FWR type, surgical repair, and Killip class III–IV) based on clinical significance and the need to maintain an adequate events-per-variable ratio given 56 death events among 71 CR patients. Given the high mortality rate in the FWR subgroup (38/39 deaths), near-complete separation was anticipated; this should be considered when interpreting the width of confidence intervals. To address this limitation and to incorporate event-time information, two additional sensitivity analyses were performed. First, a Cox proportional hazards model was fit using the same three covariates, with time measured from cardiac rupture diagnosis to in-hospital death and with patients surviving to discharge censored at the date of discharge (Supplementary Table S1). Ties were handled using Breslow's method. The proportional hazards assumption was assessed by examining the correlation between scaled Schoenfeld residuals and ranks of event time. Second, a Firth-penalized logistic regression was fit using iteratively reweighted least squares with profile penalized-likelihood confidence intervals, which stabilizes estimates in the presence of near-complete separation (Supplementary Table S2). Kaplan–Meier survival curves were constructed by rupture subtype to illustrate the temporal pattern of in-hospital mortality. Time-to-event was defined as days from rupture diagnosis to in-hospital death; patients who survived to discharge were censored at the date of discharge. Because discharge represents an informative censoring event rather than random loss to follow-up, the KM estimates should be interpreted as descriptive illustrations of the temporal mortality pattern. Survival differences were assessed using the log-rank test.

All statistical tests were two-sided, with *P* < 0.05 considered statistically significant. Analyses were performed using Python 3.12 with the scipy, scikit-learn, and statsmodels packages.

### 2.6 Ethical statement

This study was conducted in accordance with the Declaration of Helsinki. The study protocol was reviewed and approved by the Ethics Committee of Wuhan Asia General Hospital (Approval No. WAGHMEC-KY-2026002). Given the retrospective design and the use of de-identified data, the requirement for written informed consent was waived by the ethics committee.

## 3 Results

### 3.1 Study population

**Figure 1:**
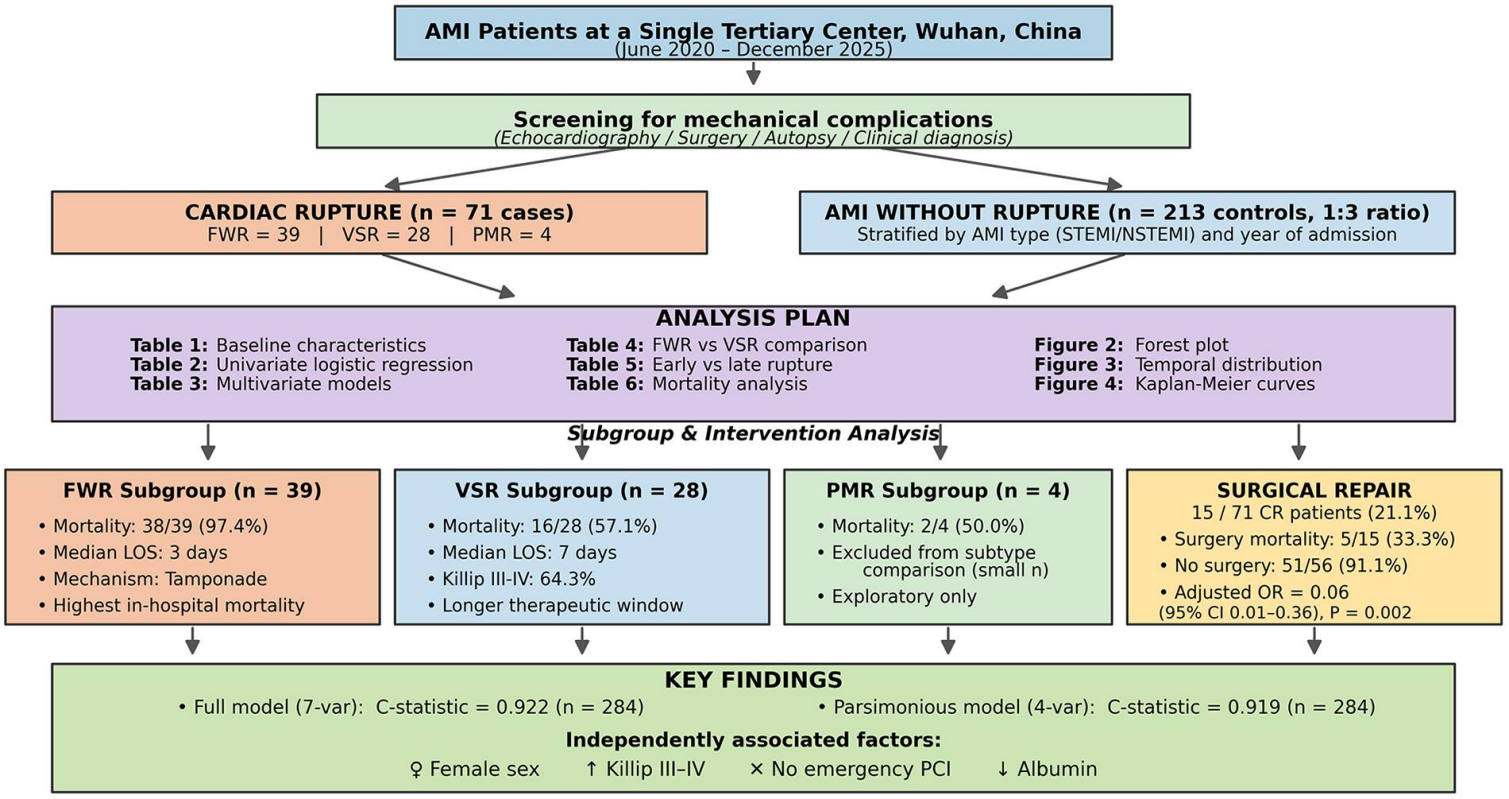
Study flow diagram. A total of 71 unique patients with cardiac rupture were identified from June 2020 to December 2025. FWR was the most common type (54.9%), followed by VSR (39.4%) and PMR (5.6%). After applying inclusion and exclusion criteria, 71 CR cases and 213 AMI controls without mechanical complications were included in the final case-control analysis.

During the study period from June 2020 to December 2025, a total of 71 unique patients with cardiac rupture were identified. FWR was the most common subtype (*n* = 39, 54.9%), followed by VSR (*n* = 28, 39.4%) and PMR (*n* = 4, 5.6%). A control group of 213 AMI patients without mechanical complications was randomly selected from the same period at a 1:3 case-to-control ratio, stratified by AMI type (STEMI vs. NSTEMI). The overall in-hospital mortality of the CR group was 78.9% (56/71). The study flow diagram is shown in Figure 1.

### 3.2 Baseline characteristics

The baseline characteristics of the CR and control groups are summarized in Table 1. Patients in the CR group were significantly older (69.6 ± 8.5 vs. 59.5 ± 11.7 years, *P* < 0.001) and more likely to be female (40.8% vs. 12.2%, *P* < 0.001). Hypertension was more prevalent in the CR group (67.6% vs. 51.2%, *P* = 0.016), while smoking was significantly less frequent (32.9% vs. 58.7%, *P* < 0.001). There was no significant difference in the prevalence of diabetes (28.2% vs. 25.4%, *P* = 0.640) or AMI type (STEMI: 97.2% vs. 95.3%, *P* = 0.736).

**Table 1:** Comparison of baseline characteristics between the cardiac rupture group (*n* = 71) and the control group (*n* = 213).

**Variable**	**CR Group** **(*n* = 71)**	**Control** **(*n* = 213)**	***P* value**
**Demographics & Comorbidities**	–	–	–
Age, years	69.59 ± 8.50	59.51 ± 11.72	**<0** **.** **001**
Female sex	29 (40.8%)	26 (12.2%)	**<0** **.** **001**
Hypertension	48 (67.6%)	109 (51.2%)	**0** **.** **016**
Diabetes	20 (28.2%)	54 (25.4%)	0.640
Smoking	23 (32.9%)	125 (58.7%)	**<0** **.** **001**
SBP, mmHg	109.37 ± 20.17	134.67 ± 26.84	**<0** **.** **001**
DBP, mmHg	65.26 ± 12.69	77.13 ± 13.77	**<0** **.** **001**
**Clinical Presentation**	–	–	–
Heart rate, bpm	85.00 (74.00–99.00)	80.00 (71.00–89.00)	**0** **.** **034**
Killip III-IV	32 (45.1%)	17 (8.0%)	**<0** **.** **001**
STEMI	69 (97.2%)	203 (95.3%)	0.736
WBC, x10^9/L	11.70 (9.26–14.78)	9.94 (7.92–12.06)	**<0** **.** **001**
**Laboratory Tests**	–	–	-
Neutrophil, x10^9/L	9.09 (6.35–12.02)	7.30 (4.91–9.56)	**<0** **.** **001**
Lymphocyte, x10^9/L	1.47 (0.96–1.89)	1.64 (1.19–2.39)	**0** **.** **010**
Monocyte, x10^9/L	0.88 (0.66–1.27)	0.60 (0.48–0.78)	**<0** **.** **001**
Platelet, x10^9/L	217.00 (175.00–261.00)	221.00 (189.00–259.00)	0.593
Hemoglobin, g/L	124.00 (114.00–141.00)	148.00 (134.00–161.00)	**<0** **.** **001**
Albumin, g/L	36.10 (33.20–39.80)	43.11 (40.50–45.50)	**<0** **.** **001**
Creatinine, umol/L	99.00 (77.75–149.50)	82.00 (69.00–101.00)	**<0** **.** **001**
eGFR, mL/min/1.73 m^2^	56.00 (40.00–69.25)	86.00 (65.00–96.00)	**<0** **.** **001**
Glucose, mmol/L	8.74 (7.11–13.41)	7.79 (6.44–10.07)	**0** **.** **003**
cTnT, ng/L	2,174.00 (651.00–3,275.00)	1,443.50 (431.00–3,810.00)	0.380
NT-proBNP, pg/mL	3,826.00 (2,149.00–9,550.25)	265.50 (97.97–1,051.50)	**<0** **.** **001**
D-Dimer, ug/mL	1.92 (0.86–3.61)	0.33 (0.22–0.67)	**<0** **.** **001**
hs-CRP, mg/L	36.60 (9.96–84.33)	4.32 (1.75–14.04)	**<0** **.** **001**
LVEF, %	43.50 (40.00–46.75)	45.00 (42.00–50.00)	**<0** **.** **001**
LVEF <40%	16 (22.5%)	10 (4.7%)	**<0** **.** **001**
Onset-to-door, hours	48.00 (24.00–96.00)	5.00 (3.00–11.62)	**<0** **.** **001**
No emergency PCI	61 (85.9%)	57 (26.8%)	**<0** **.** **001**
DAPT	63 (88.7%)	212 (99.5%)	**<0** **.** **001**
**Treatment Before Rupture Diagnosis** ^a^	–	–	–
ACEI/ARB	0 (0.0%)	57 (26.8%)	**<0** **.** **001**
Beta-blocker	31 (43.7%)	173 (81.2%)	**<0** **.** **001**
IABP	36 (50.7%)	26 (12.2%)	**<0** **.** **001**
ECMO	7 (9.9%)	7 (3.3%)	0.050

Data are presented as mean ± SD, median (IQR), or *n* (%). CR, cardiac rupture; SBP, systolic blood pressure; eGFR, estimated glomerular filtration rate; cTnT, cardiac troponin T; NT-proBNP, N-terminal pro-B-type natriuretic peptide; LVEF, left ventricular ejection fraction; PCI, percutaneous coronary intervention; DAPT, dual antiplatelet therapy; IABP, intra-aortic balloon pump; ECMO, extracorporeal membrane oxygenation.

Bold values indicate statistical significance (*P* < 0.05).

a Treatment variables reflect exposure status before the diagnosis of cardiac rupture in the CR group and during the corresponding hospitalization period in controls. These variables may represent downstream consequences of delayed presentation and should not be interpreted as independent upstream contributors (see Discussion).

Patients with CR presented with more severe hemodynamic compromise, as evidenced by lower systolic blood pressure (109.4 ± 20.2 vs. 134.7 ± 26.8 mmHg, *P* < 0.001), lower diastolic blood pressure (65.3 ± 12.7 vs. 77.1 ± 13.8 mmHg, *P* < 0.001), and a substantially higher proportion of Killip class III–IV (45.1% vs. 8.0%, *P* < 0.001).

Laboratory findings revealed a distinct profile in the CR group: lower serum albumin (36.1 vs. 43.1 g/L, *P* < 0.001), lower hemoglobin (124 vs. 148 g/L, *P* < 0.001), impaired renal function (eGFR 56 vs. 86 mL/min/1.73 m^2^, *P* < 0.001), and markedly elevated NT-proBNP (3,826 vs. 266 pg/mL, *P* < 0.001) and D-dimer (1.92 vs. 0.33 μg/mL, *P* < 0.001). Inflammatory markers were also elevated, including higher white blood cell count (11.70 vs. 9.94 × 10^9^/L, *P* < 0.001), neutrophil count (9.09 vs. 7.30 × 10^9^/L, *P* < 0.001), and hs-CRP (36.6 vs. 4.3 mg/L, *P* < 0.001). Admission cardiac troponin T did not differ significantly between groups (*P* = 0.380).

Notably, onset-to-door time was dramatically longer in the CR group (median 48 vs. 5 h, *P* < 0.001), and the rate of emergency PCI was profoundly lower (14.1% vs. 73.2%, *P* < 0.001). Guideline-directed medications before rupture diagnosis were also less frequently recorded in the CR group, including DAPT (88.7% vs. 99.5%, *P* < 0.001), beta-blockers (43.7% vs. 81.2%, *P* < 0.001), and ACEI/ARB (0% vs. 26.8%, *P* < 0.001). IABP use was more common in the CR group (50.7% vs. 12.2%, *P* < 0.001).

### 3.3 Risk factors for cardiac rupture

Univariate logistic regression analysis identified 22 of 26 candidate variables as significantly associated with cardiac rupture (Table 2). The factors with the strongest associations were absence of emergency PCI (OR=16.69, 95% CI 8.01–34.78, *P* < 0.001), Killip class III–IV (OR = 9.47, 95% CI 4.79–18.71, *P* < 0.001), LVEF < 40% (OR = 5.91, 95% CI 2.54–13.75, *P* < 0.001), and female sex (OR = 4.97, 95% CI 2.66–9.29, *P* < 0.001). The strongest protective associations were observed for higher serum albumin (OR = 0.75 per g/L, *P* < 0.001), higher hemoglobin (OR = 0.97 per g/L, *P* < 0.001), and smoking (OR = 0.34, *P* < 0.001).

**Table 2:** Univariate logistic regression analysis for factors associated with cardiac rupture (*N* = 284).

**Variable**	**OR**	**95% CI**	***P* value**
Age (per year)	1.100	1.065–1.136	**<0.001**
Female sex	4.966	2.655–9.290	**<0.001**
Hypertension	1.988	1.130–3.497	**0.017**
Diabetes	1.155	0.633–2.110	0.639
Smoking	0.344	0.195–0.608	**<0.001**
Killip III-IV	9.468	4.790–18.713	**<0.001**
No emergency PCI	16.690	8.009–34.778	**<0.001**
LVEF <40%	5.909	2.539–13.748	**<0.001**
Albumin (per g/L)	0.752	0.696–0.813	**<0.001**
Hemoglobin (per g/L)	0.965	0.952–0.978	**<0.001**
Creatinine (per umol/L)	1.004	1.000–1.007	**0.032**
eGFR (per unit)	0.963	0.952–0.975	**<0.001**
Glucose (per mmol/L)	1.099	1.042–1.160	**<0.001**
cTnT (per 100 ng/L)	1.001	0.998–1.005	0.459
NT-proBNP (per 100 pg/mL)	1.023	1.014–1.032	**<0.001**
D-Dimer (per ug/mL)	1.236	1.080–1.415	**0.002**
hs-CRP (per mg/L)	1.018	1.010–1.026	**<0.001**
WBC (per 10^9/L)	1.133	1.058–1.213	**<0.001**
Neutrophil (per 10^9/L)	1.145	1.067–1.230	**<0.001**
Onset-to-door (per h)	1.011	1.006–1.015	**<0.001**
SBP (per mmHg)	0.957	0.944–0.971	**<0.001**
DBP (per mmHg)	0.933	0.911–0.956	**<0.001**
Heart rate (per bpm)	1.013	0.999–1.028	0.073
IABP use	7.396	3.978–13.753	**<0.001**
DAPT use	0.037	0.005–0.303	**0.002**
Beta-blocker use	0.179	0.100–0.320	**<0.001**

OR, odds ratio; CI, confidence interval. NT-proBNP is expressed per 100 pg/mL increase. IABP was excluded from multivariate analysis as it likely represents a consequence of hemodynamic deterioration. ACEI/ARB use was excluded from univariate analysis due to complete separation (0% in the CR group).

Bold values indicate statistical significance (*P* < 0.05).

In the full multivariable model incorporating seven variables (*n* = 284; Table 3A), four variables retained independent significance: absence of emergency PCI (OR = 7.00, 95% CI 2.79–17.57), Killip class III–IV (OR = 5.75, 95% CI 2.14–15.41), female sex (OR = 3.09, 95% CI 1.23–7.79), and serum albumin (OR = 0.86 per g/L, 95% CI 0.77–0.95). The model demonstrated high discrimination (C-statistic = 0.922), although apparent discrimination may be inflated in case-control designs with substantial baseline differences between groups, and acceptable calibration (Hosmer-Lemeshow *P* = 0.667).

**Table 3A:** Full multivariate model (*n* = 284, 7 variables, EPV = 10.1).

**Variable**	**OR**	**95% CI**	***P* value**
Age (per year)	1.038	0.994–1.084	0.090
Female sex	3.092	1.227–7.792	**0** **.** **017**
Killip III-IV	5.745	2.143–15.405	**<0** **.** **001**
No emergency PCI	7.003	2.792–17.571	**<0** **.** **001**
Albumin (per g/L)	0.856	0.774–0.947	**0** **.** **003**
Hemoglobin (per g/L)	0.999	0.977–1.021	0.912
Onset-to-door (per hour)	1.002	0.998–1.006	0.260

Bold values indicate statistical significance (*P* < 0.05).

C-statistic (AUC) = 0.9224. Hosmer-Lemeshow test: *χ*^2^ = 5.82, *P* = 0.667, indicating good calibration.

**Figure 2:**
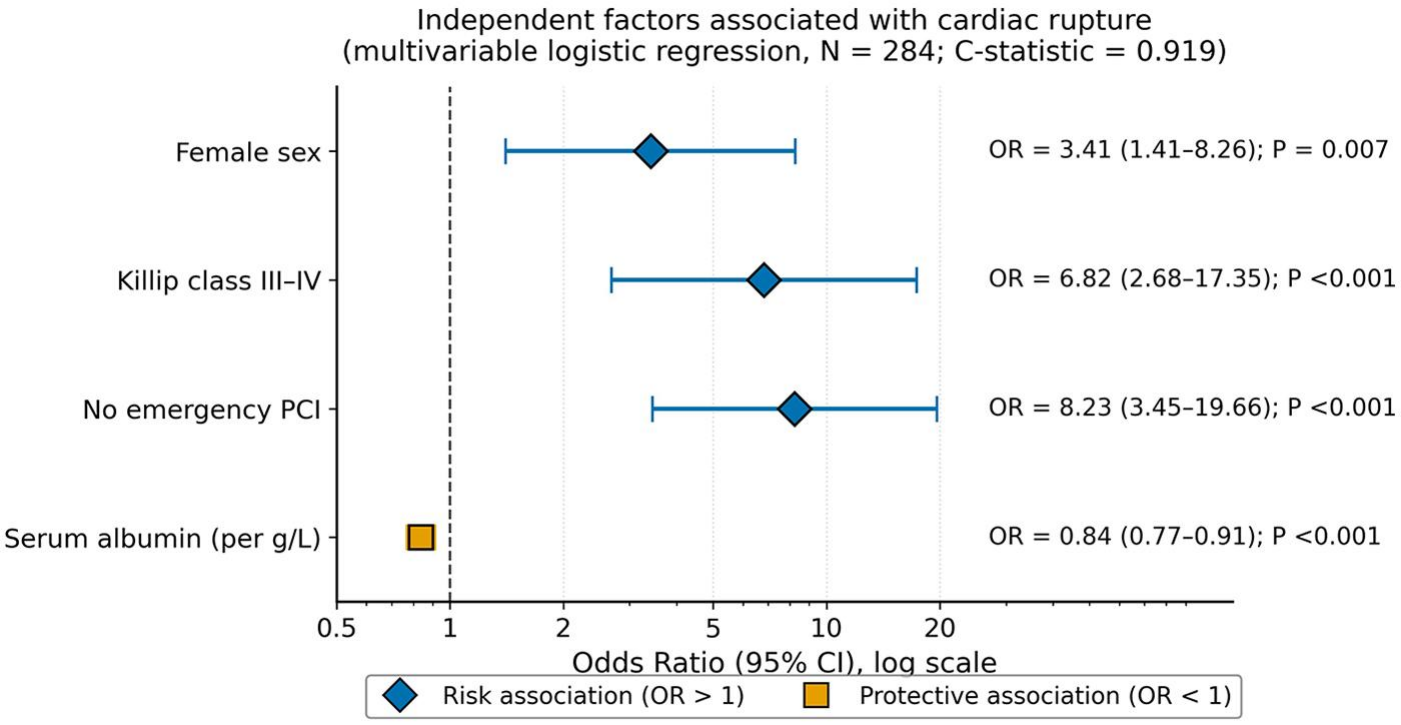
Forest plot of the multivariate logistic regression model. Diamond markers represent point estimates of odds ratios; horizontal bars represent 95% CIs. Blue diamonds = factors associated with increased risk (OR > 1, *P* < 0.05); orange squares = factors associated with lower risk (OR < 1, *P* < 0.05). Four variables achieved independent significance: absence of emergency PCI, Killip class III–IV, female sex, and serum albumin. C-statistic = 0.922.

A parsimonious model retaining only the four independent predictors (Table 3B) yielded comparable discrimination (C-statistic = 0.919). In this model, absence of emergency PCI remained the factor most strongly associated with CR, likely reflecting delayed presentation and reduced reperfusion opportunity at the system level rather than a directly modifiable individual-level exposure (OR = 8.23, 95% CI 3.45–19.66, *P* < 0.001), followed by Killip class III–IV (OR = 6.82, 95% CI 2.68–17.35, *P* < 0.001), female sex (OR = 3.41, 95% CI 1.41–8.26, *P* = 0.007), and lower serum albumin (OR = 0.84 per g/L, 95% CI 0.77–0.91, *P* < 0.001). The forest plot of the multivariable model is presented in Figure 2.

**Table 3B:** Parsimonious model (*n* = 284, 4 independent predictors, C-statistic = 0.9185).

**Variable**	**OR**	**95% CI**	***P* value**
Female sex	3.408	1.405–8.264	**0** **.** **007**
Killip III-IV	6.821	2.682–17.349	**<0** **.** **001**
No emergency PCI	8.232	3.448–19.656	**<0** **.** **001**
Albumin (per g/L)	0.836	0.767–0.912	**<0** **.** **001**

Bold values indicate statistical significance (*P* < 0.05).

### 3.4 Comparison of FWR and VSR

The clinical characteristics of FWR (*n* = 39) and VSR (*n* = 28) are compared in Table 4. PMR cases (*n* = 4) were excluded from this comparison due to the extremely small sample size and are described separately in the supplementary material. Given the modest sample size of the VSR group and the descriptive nature of the PMR subgroup, the subtype comparisons should be considered exploratory and hypothesis-generating.

**Table 4:** Clinical characteristics comparison between FWR (*n* = 39) and VSR (*n* = 28). PMR (*n* = 4) excluded due to insufficient sample size.

**Variable**	**FWR (*n* = 39)**	**VSR (*n* = 28)**	***P* value**
Age	69.36 ± 7.87	69.54 ± 9.35	0.933
Female	16 (41.0%)	12 (42.9%)	0.881
Heart rate	83.38 ± 19.47	96.54 ± 19.28	**0** **.** **008**
SBP	110.00 (98.00–131.00)	100.00 (93.50–109.00)	0.074
Killip III-IV	12 (30.8%)	18 (64.3%)	**0** **.** **007**
WBC	12.39 ± 4.86	12.53 ± 4.84	0.911
Neutrophil	8.58 (6.21–12.33)	9.81 (6.79–10.85)	0.556
Lymphocyte	1.43 (0.94–1.96)	1.54 (1.17–1.82)	0.499
Hemoglobin	125.00 (115.50–139.00)	124.00 (113.00–151.25)	0.979
Albumin	37.50 (33.80–40.55)	35.50 (33.20–38.60)	0.255
Creatinine	90.00 (72.00–138.50)	110.00 (89.50–170.25)	**0** **.** **037**
Glucose	8.51 (6.60–11.13)	10.70 (8.14–17.03)	**0** **.** **038**
cTnT	1,946.00 (850.00–3,011.00)	2,500.00 (589.75–3,713.25)	0.729
NT-proBNP	2,315.50 (969.25–4,943.25)	8,836.50 (3,395.00–14,826.75)	**<0** **.** **001**
D-Dimer	1.74 (0.51–3.81)	2.38 (1.20–3.79)	0.213
LVEF	43.50 (40.00–47.00)	43.00 (40.00–45.00)	0.724
No emergency PCI	34 (87.2%)	25 (89.3%)	1.000
Onset-to-door	48.00 (21.00–96.00)	72.00 (24.00–180.00)	0.134
Onset-to-rupture, d	5.00 (3.00–7.50)	3.00 (1.00–6.25)	0.078
In-hospital death	38 (97.4%)	16 (57.1%)	**<0** **.** **001**
Length of stay, d	3.00 (2.00–6.00)	7.00 (3.25–26.25)	**0** **.** **010**

Bold values indicate statistical significance (*P* < 0.05).

The two subtypes did not differ significantly in age (69.4 ± 7.9 vs. 69.5 ± 9.4 years, *P* = 0.933), sex distribution (female: 41.0% vs. 42.9%, *P* = 0.881), or emergency PCI rates (12.8% vs. 10.7%, *P* = 1.000). However, VSR patients demonstrated more pronounced hemodynamic deterioration, with a significantly higher heart rate (96.5 ± 19.3 vs. 83.4 ± 19.5 bpm, *P* = 0.008), greater prevalence of Killip class III–IV (64.3% vs. 30.8%, *P* = 0.007), and markedly higher NT-proBNP levels (median 8,837 vs. 2,316 pg/mL, *P* < 0.001). VSR patients also had higher serum creatinine (110 vs. 90 μmol/L, *P* = 0.037) and blood glucose (10.7 vs. 8.5 mmol/L, *P* = 0.038).

Despite the more severe hemodynamic profile in the VSR group, in-hospital mortality was significantly higher in FWR patients (97.4% vs. 57.1%, *P* < 0.001). Correspondingly, FWR patients had a shorter length of hospital stay (median 3 vs. 7 days, *P* = 0.010), reflecting the rapidly fatal course of free wall rupture. The onset-to-rupture interval showed a non-significant trend toward earlier occurrence in VSR (median 3 vs. 5 days, *P* = 0.078).

### 3.5 Temporal distribution of cardiac rupture

**Figure 3:**
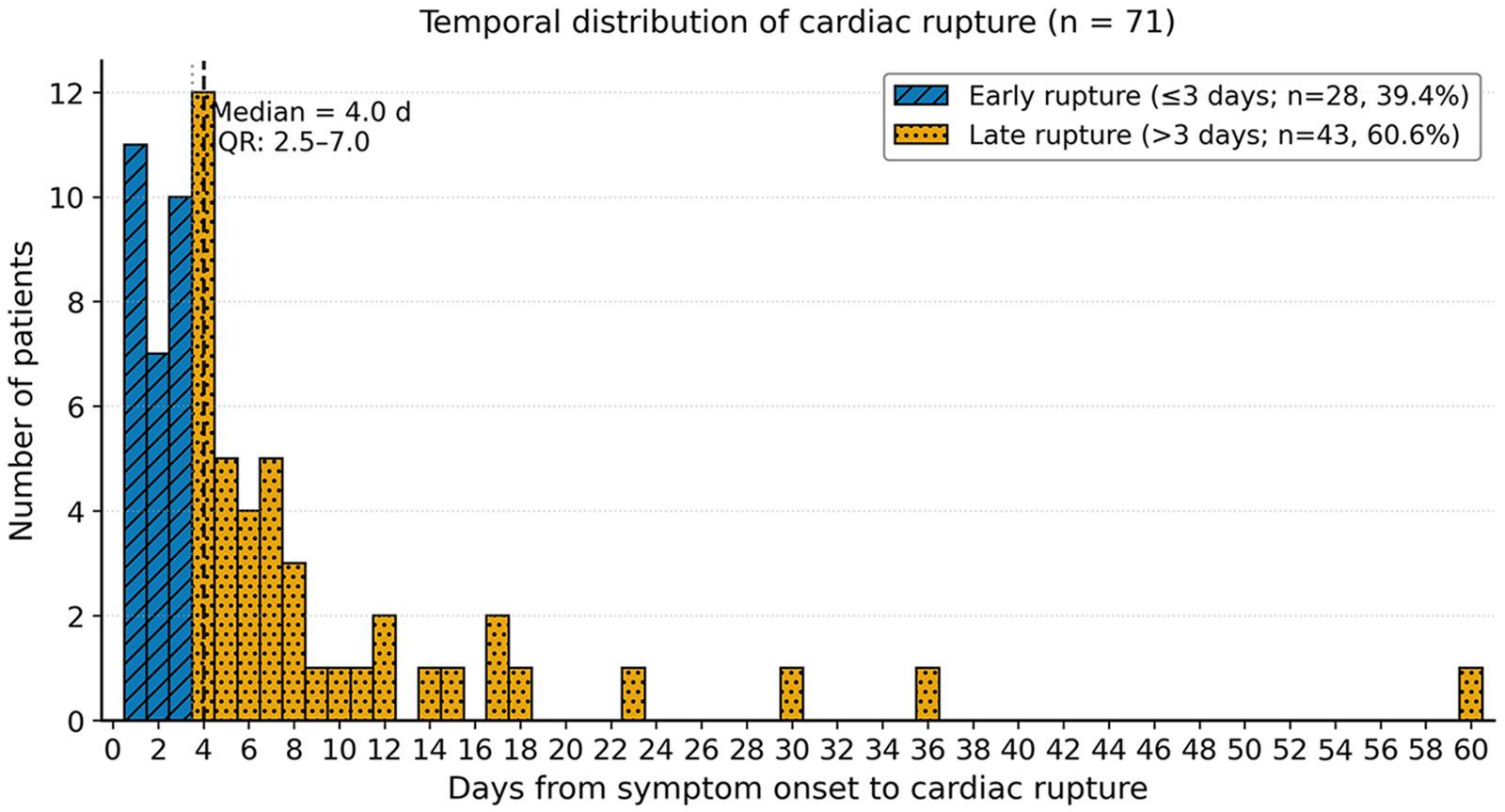
Temporal distribution of cardiac rupture after AMI onset. Median time: 4.0 days (IQR 2.5–7.0). Blue (hatched) = early rupture (≤3 days; *n* = 28, 39.4%); orange (dotted) = late rupture (>3 days; *n* = 43, 60.6%). The bimodal appearance is compatible with two temporal patterns: earlier events during the acute transmural phase and later events during the inflammatory-remodeling phase; the observational design does not establish distinct mechanisms.

The median time from AMI onset to cardiac rupture was 4.0 days (IQR 2.5–7.0). Twenty-eight patients (39.4%) experienced early rupture (≤3 days) and 43 (60.6%) experienced late rupture (>3 days). The temporal distribution is shown in Figure 3.

Comparisons between early and late rupture groups are presented in Table 5. Early rupture patients exhibited a more intense acute inflammatory response, with higher white blood cell counts (12.42 vs. 10.97 × 10^9^/L, *P* = 0.026), higher neutrophil counts (9.84 vs. 8.27 × 10^9^/L, *P* = 0.031), and higher admission cTnT levels (median 2,837 vs. 1,856 ng/L, *P* = 0.015), suggesting a larger initial infarct burden. In contrast, late rupture patients had lower serum albumin (35.5 vs. 38.2 g/L, *P* = 0.023) and markedly longer onset-to-door times (median 96 vs. 24 h, *P* < 0.001). No significant differences were observed in age, sex, Killip class, LVEF, or in-hospital mortality between the two groups.

**Table 5:** Comparison between early rupture (≤3 days, *n* = 28) and late rupture (>3 days, *n* = 43).

**Variable**	**Early ≤3d (*n* = 28)**	**Late >3d (*n* = 43)**	***P* value**
Age	68.50 ± 9.18	70.30 ± 8.05	0.386
Female	12 (42.9%)	17 (39.5%)	0.781
Heart rate	90.57 ± 20.79	84.44 ± 22.21	0.248
SBP	104.19 ± 18.13	112.63 ± 20.90	0.088
Killip III-IV	15 (53.6%)	17 (39.5%)	0.245
WBC	12.42 (11.01–16.15)	10.97 (8.16–14.10)	**0** **.** **026**
Neutrophil	9.84 (8.87–13.32)	8.27 (6.14–11.04)	**0** **.** **031**
Lymphocyte	1.53 (1.31–2.02)	1.36 (0.93–1.71)	0.071
Hemoglobin	132.00 (120.00–155.50)	122.50 (113.00–132.00)	0.050
Albumin	38.20 (35.45–41.70)	35.45 (32.10–38.10)	**0** **.** **023**
Creatinine	97.00 (82.00–173.00)	101.00 (72.00–139.00)	0.319
Glucose	9.10 (8.28–17.33)	8.62 (6.72–12.20)	0.114
cTnT	2,837.00 (2,081.50–3,974.50)	1,856.00 (549.50–2,911.50)	**0** **.** **015**
NT-proBNP	3,758.00 (2,232.50–9,105.00)	3,894.00 (1,713.00–10,717.00)	0.878
LVEF	42.00 (39.50–45.25)	44.50 (40.00–47.00)	0.418
No emergency PCI	25 (89.3%)	36 (83.7%)	0.730
Onset-to-door	24.00 (14.25–48.00)	96.00 (48.00–168.00)	**<0** **.** **001**
In-hospital death	24 (85.7%)	32 (74.4%)	0.254

Bold values indicate statistical significance (*P* < 0.05).

### 3.6 In-Hospital mortality and predictors

**Figure 4:**
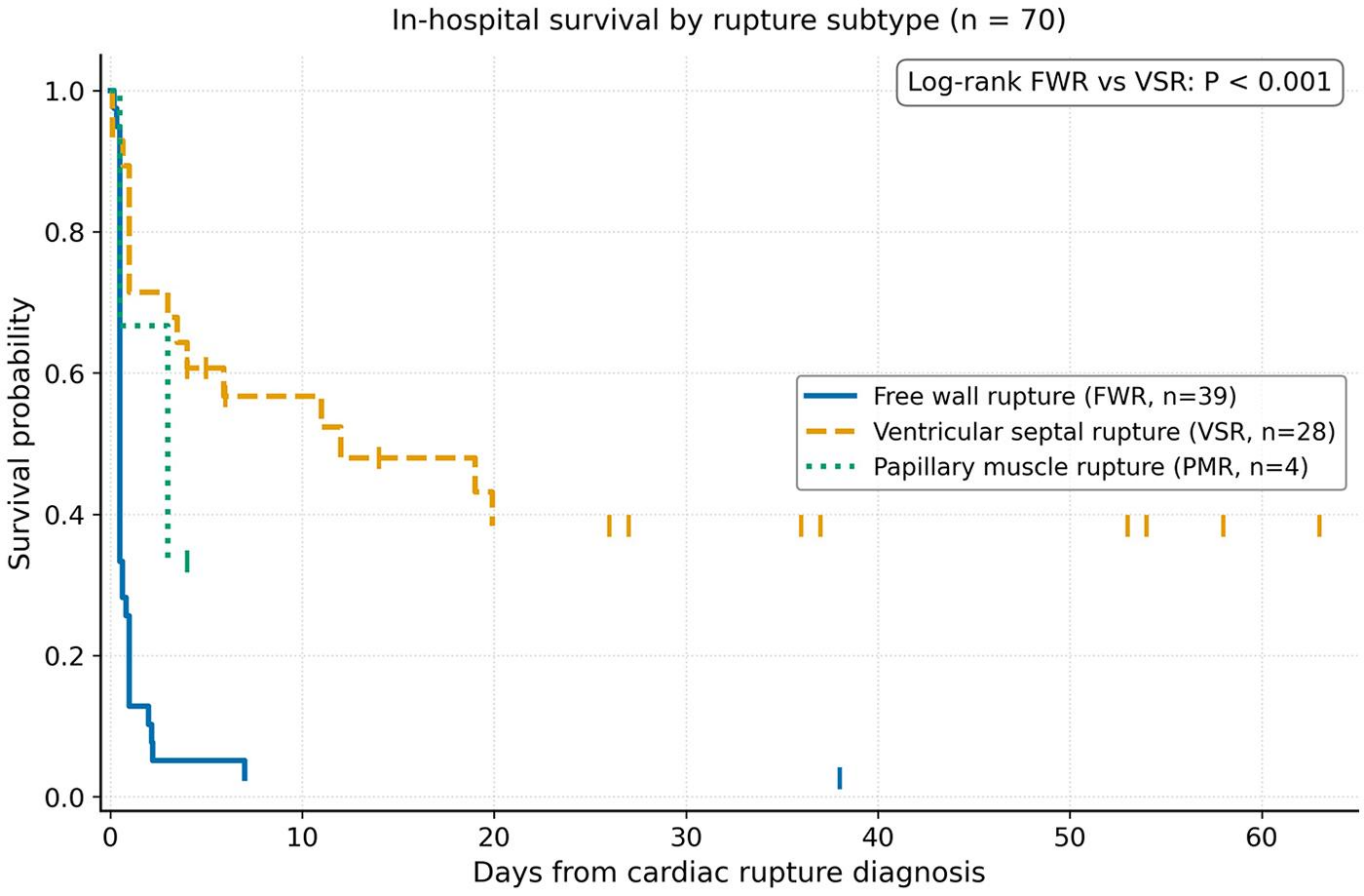
Kaplan–meier survival curves by rupture type. FWR (solid blue) showed near-complete mortality (97.4%) within the first week, reflecting the immediately lethal nature of free wall rupture. VSR (dashed orange) demonstrated a more gradual decline with 42.9% surviving to discharge. PMR (dotted teal) showed intermediate survival. Log-rank test: FWR vs. VSR *P* < 0.001. Tick marks indicate censored observations (survival to discharge).

Overall in-hospital mortality in the CR group was 78.9% (56 of 71). Among 71 CR patients, 15 (21.1%) underwent surgical repair; the crude in-hospital mortality rate was substantially lower in the surgical group (33.3%, 5/15) compared with the non-surgical group (91.1%, 51/56). Mortality differed substantially by rupture type: 97.4% (38/39) for FWR, 57.1% (16/28) for VSR, and 50.0% (2/4) for PMR. Kaplan–Meier survival curves demonstrated a significant difference in in-hospital survival across rupture types (log-rank *P* < 0.001; Figure 4). FWR patients exhibited near-complete mortality within the first week, whereas VSR patients showed a more gradual decline with a longer therapeutic window.

Univariate logistic regression for in-hospital mortality (Table 6A) identified FWR type (OR = 29.53, 95% CI 3.60–242.21, *P* = 0.002) and pericardiocentesis (OR = 20.07, *P* = 0.005) as strongly associated with death. Surgical repair was the only factor significantly associated with survival (OR = 0.049, 95% CI 0.01–0.20, *P* < 0.001). Absence of emergency PCI was also associated with increased mortality (OR = 5.11, *P* = 0.024).

**Table 6A:** Univariate predictors of in-hospital mortality within the cardiac rupture group (56/71 deaths, 78.9%).

**Variable**	**OR**	**95% CI**	***P* value**
FWR type	29.534	3.601–242.210	**0** **.** **002**
Surgical repair	0.049	0.012–0.202	**<0** **.** **001**
Pericardiocentesis	20.073	2.466–163.418	**0** **.** **005**
Killip III-IV	0.219	0.062–0.774	**0** **.** **018**
Age	1.019	0.953–1.090	0.579
Albumin	1.044	0.959–1.137	0.319
Hemoglobin	0.993	0.970–1.017	0.575
No emergency PCI	5.112	1.244–20.998	**0** **.** **024**
Female	2.218	0.629–7.819	0.215
LVEF	1.009	0.941–1.082	0.794

Bold values indicate statistical significance (*P* < 0.05).

In multivariable analysis (Table 6B), FWR type remained independently associated with mortality (OR = 12.50, 95% CI 1.30–119.97, *P* = 0.029), and surgical repair remained independently associated with lower mortality (OR = 0.06, 95% CI 0.01–0.36, *P* = 0.002). Killip class III–IV did not retain statistical significance after adjustment (OR = 0.26, 95% CI 0.04–1.60, *P* = 0.146). Kaplan–Meier analysis confirmed the markedly worse survival of FWR compared with VSR and PMR (log-rank *P* < 0.001; Figure 4).

**Table 6B:** Multivariate mortality analysis (*n* = 71).

**Variable**	**OR**	**95% CI**	***P* value**
FWR type	12.495	1.301–119.967	**0** **.** **029**
Surgical repair	0.060	0.010–0.361	**0** **.** **002**
Killip III-IV	0.260	0.042–1.602	0.146

Bold values indicate statistical significance (*P* < 0.05).

## 4 Discussion

In this single-center case-control study from central China, we systematically analyzed the clinical characteristics and factors associated with cardiac rupture following AMI. Four variables were independently associated with CR: absence of emergency PCI, Killip class III–IV, female sex, and low serum albumin. These four independently associated factors are consistent with those reported in recent meta-analyses (3, 5), thereby providing external validation of these associations in a previously underrepresented population from central China. The multivariable model demonstrated high discrimination (C-statistic = 0.922), although this estimate should be interpreted with caution given that apparent discrimination in case-control studies may be inflated by large baseline differences between cases and controls; external validation in a prospective cohort is essential before the model is used for clinical prediction. Beyond confirmatory analysis, this study offers two additional contributions that are less well addressed in the existing literature: (1) a direct head-to-head comparison of FWR and VSR within the same cohort, revealing distinct hemodynamic profiles despite similar demographics; and (2) a temporal characterization of early vs. late rupture, suggesting different pathophysiological patterns that may inform surveillance strategies. Additionally, surgical repair was associated with improved survival among selected patients, while acknowledging the likelihood of survivor and indication bias.

### 4.1 Absence of emergency PCI as a system-level marker of delayed reperfusion

Absence of emergency PCI emerged as the factor most strongly associated with CR in our analysis (OR = 8.23). In our cohort, this variable is closely linked to markedly delayed presentation (median onset-to-door time 48 vs. 5 h) and should therefore be understood as a system-level marker of delayed reperfusion opportunity rather than a directly modifiable individual-level exposure. This interpretation is consistent with a large body of evidence supporting the protective role of timely reperfusion against mechanical complications (5, 8–12). A recent systematic review and meta-analysis of risk prediction models for CR confirmed that PCI status was among the most robust correlates across studies (5). Early reperfusion limits infarct expansion, attenuates transmural necrosis, and reduces the inflammatory cascade responsible for extracellular matrix degradation—all of which are central to the pathogenesis of myocardial rupture (1, 7). In our cohort, only 14.1% of CR patients underwent emergency PCI compared to 73.2% of controls, and the median onset-to-door time was dramatically longer in the CR group (48 vs. 5 h). These findings reinforce the critical importance of minimizing time to reperfusion, particularly in regions where delayed presentation remains prevalent and contemporary guidelines continue to prioritize early revascularization for patients with ACS and STEMI (12). Of note, onset-to-door time itself did not retain significance in the multivariable model, likely because its effect was largely captured by the PCI variable—patients who presented late were inherently less likely to receive emergency PCI.

Notably, several treatment variables showed marked between-group differences, including ACEI/ARB use (0% vs. 26.8%), beta-blocker use (43.7% vs. 81.2%), and DAPT (88.7% vs. 99.5%). These differences should be interpreted with caution, as they likely reflect the consequences of delayed presentation rather than independent upstream contributors. In our cohort, the median onset-to-door time was 48 h in the CR group compared with 5 h in controls, meaning that CR patients had substantially less opportunity to receive guideline-directed medical therapy before rupture occurred. Similarly, IABP use was more prevalent in the CR group (50.7% vs. 12.2%), most likely as a response to hemodynamic deterioration rather than a predisposing factor. For these reasons, medication variables and mechanical circulatory support were not included in the primary multivariable model. The pattern most consistent with our data is that delayed presentation is associated with reduced opportunity for emergency PCI and guideline-directed pharmacotherapy, which in turn correlates with higher CR risk. Future studies with time-stamped medication data and formal mediation analysis could help disentangle these relationships.

### 4.2 Killip class III–IV and hemodynamic instability

Killip class III–IV was the second most strongly associated factor in our study (OR = 6.82). This finding aligns with the recent systematic review and meta-analysis of CR prediction models, which identified higher Killip grade as a consistently associated factor (5). Hemodynamic instability in the setting of AMI reflects extensive myocardial injury, elevated ventricular wall stress, and neurohumoral activation, all of which may predispose to mechanical failure of the infarcted wall. In our cohort, 45.1% of CR patients presented with Killip class III–IV compared to only 8.0% of controls, underscoring the prognostic importance of early hemodynamic assessment. Clinically, patients presenting with advanced heart failure after AMI should be regarded as high-risk for mechanical complications and warrant intensified monitoring, including serial echocardiography and timely consideration of hemodynamic support and revascularization strategies in keeping with contemporary ACS and cardiogenic shock literature (12–14).

### 4.3 Female Sex as an independent risk factor

Female sex was independently associated with a three-fold increased risk of CR (OR = 3.41), consistent with the pooled estimate from a meta-analysis of 16 studies (pooled OR = 2.72) (3). Several mechanisms have been proposed to explain this sex disparity. First, women tend to present with AMI at an older age, and experimental data suggest that sex-related differences in the post-infarction inflammatory and matrix-remodeling response may contribute to rupture susceptibility after infarction (15). Second, women are more likely to experience atypical symptom presentations, leading to delayed diagnosis and treatment—a pattern reflected in our data, where the CR group had significantly longer onset-to-door times. Third, female patients may have smaller ventricular chamber dimensions and thinner walls, which could render the myocardium more susceptible to rupture under equivalent hemodynamic stress (7). Contemporary clinical series have likewise continued to identify female sex and older age as prominent correlates of CR in both general AMI cohorts and PCI-era STEMI populations (4, 8, 9). In our cohort, women constituted 40.8% of CR cases but only 12.2% of controls, highlighting the need for heightened clinical vigilance in female AMI patients.

### 4.4 Low serum albumin as a marker of vulnerability

Serum albumin was the only continuous laboratory variable that retained independent significance in the multivariable model (OR = 0.84 per g/L increase). Each 1 g/L increase in albumin was associated with a 16% reduction in the odds of CR. This finding corroborates the report by Gao et al. (2024), who identified albumin as an independently associated factor in their single-center study (4). Hypoalbuminemia in the context of AMI reflects a convergence of several pathological processes, including systemic inflammation, nutritional depletion, and capillary leakage due to endothelial dysfunction (16). Additionally, albumin possesses antioxidant, anticoagulant, and antiplatelet properties; its deficiency may therefore exacerbate oxidative damage and platelet activation within the healing myocardium (16, 17). The clinical implication is that serum albumin measured at admission may serve as a readily available marker to identify patients at heightened risk for CR, and that nutritional support strategies in the acute phase of AMI deserve further investigation.

### 4.5 Distinct clinical profiles of FWR and VSR

An important contribution of this study is the direct comparison of FWR and VSR within the same cohort. Despite similar age, sex distribution, and reperfusion status, the two subtypes showed clearly different clinical and hemodynamic profiles. VSR was characterized by more severe heart failure at presentation, reflected by higher Killip class, faster heart rate, and markedly higher NT-proBNP levels. In contrast, FWR was associated with nearly universal early in-hospital mortality and much less opportunity for successful intervention. These findings suggest that monitoring priorities and therapeutic planning may differ between rupture subtypes, with prevention and immediate recognition being especially critical for FWR and early operative evaluation remaining central for VSR and PMR, in keeping with prior subtype-focused literature (18, 19).

### 4.6 Temporal pattern of cardiac rupture

The temporal analysis revealed a median rupture time of 4.0 days (IQR 2.5–7.0), with 39.4% of cases occurring within 3 days. Early and late ruptures appeared to represent different temporal clinical patterns rather than definitively distinct biological phenotypes. Early rupture was associated with stronger inflammatory activation and more pronounced acute myocardial injury, whereas late rupture was more often accompanied by lower albumin levels and markedly delayed presentation, suggesting progressive structural vulnerability during infarct healing. These observations indicate that CR risk does not disappear after the first 72 h and that patients with delayed presentation, malnutrition, and persistent inflammatory activity may require continued surveillance throughout hospitalization.

### 4.7 Surgical repair and in-hospital survival

Among all variables analyzed, surgical repair was the only factor independently associated with lower in-hospital mortality in the CR group (OR = 0.06, *P* = 0.002), corresponding to an estimated 94% relative reduction in the odds of death. This association is clinically plausible and consistent with prior literature showing that definitive mechanical correction remains the cornerstone of treatment for selected patients with CR, particularly VSR and PMR, and that operative timing and patient selection remain major determinants of outcome (20–27). However, it should not be interpreted as a direct causal treatment effect because patients who survive long enough and remain stable enough to undergo surgery are inherently selected. A noteworthy finding in the mortality analysis was the paradoxical direction of the Killip III–IV estimate. The most likely explanation is confounding by rupture subtype: VSR patients more often presented with advanced heart failure, yet their short-term mortality was substantially lower than that of FWR. Accordingly, the Killip estimate should be interpreted cautiously and not as evidence of a protective effect of severe heart failure. Additionally, the wide confidence interval for FWR type in the multivariable mortality model (OR = 12.50, 95% CI 1.30–119.97) reflects near-complete separation in the data (38 of 39 FWR patients died), and should be interpreted as indicating the direction of association rather than a precise effect size. To address this concern, a Firth-penalized logistic regression was performed as a sensitivity analysis and produced a more stable estimate for FWR type (OR = 8.14, 95% profile CI 1.49–84.09; Supplementary Table S2), with the direction and statistical significance of all three model covariates preserved. A Cox proportional hazards analysis for time from rupture diagnosis to in-hospital death was additionally performed (FWR type: HR = 3.22, 95% CI 1.69–6.14; surgical repair: HR = 0.19, 95% CI 0.07–0.51; Supplementary Table S1) and yielded qualitatively concordant conclusions with narrower confidence intervals. Future multicenter studies with larger sample sizes will be important to further refine these estimates. Overall, the present findings support prompt surgical evaluation in potentially operable patients rather than delayed referral after irreversible deterioration has occurred.

### 4.8 Limitations

Several limitations of this study should be acknowledged. First, the single-center, retrospective design inherently limits the generalizability of our findings and is susceptible to residual confounding. Our hospital functions as a regional cardiovascular referral center, which may enrich for high-acuity AMI presentations and could therefore overestimate the incidence of mechanical complications relative to primary or community settings. The findings should be interpreted within this referral context, and multicenter validation in more representative populations will be essential before generalization. Second, the relatively small number of CR cases, particularly within the PMR subgroup, constrained statistical power for some subgroup and prognostic analyses. Third, although several treatment-related variables were analyzed, the observational design precludes causal inference regarding management strategies. In particular, the association between surgical repair and lower mortality may reflect indication bias and survivor bias in addition to true therapeutic benefit. Fourth, external validation of the risk model was not performed, and therefore the findings should be interpreted as hypothesis-generating and requiring confirmation in larger multicenter cohorts. Fifth, the control group was not matched to cases by age or sex. This was a deliberate design choice, because age and sex were prespecified candidate predictor variables in the multivariable analysis; matching on these factors would have precluded estimation of their independent associations with CR. Instead, controls were stratified by AMI type (STEMI vs. NSTEMI) and year of admission, and the resulting baseline differences in age and sex were addressed through multivariable adjustment. Nevertheless, the substantial imbalance between groups (mean age 69.6 vs. 59.5 years; female proportion 40.8% vs. 12.2%) should be considered when interpreting the adjusted estimates, and residual confounding cannot be entirely excluded. Sixth, the Kaplan–Meier survival analysis treated hospital discharge as a censoring event. Because discharge is an informative event reflecting clinical improvement rather than random loss to follow-up, this may introduce bias into the survival estimates. Competing risk methods treating discharge as a competing event would provide more appropriate estimates, but were not performed due to the small sample size. As such, the KM curves should be regarded as descriptive illustrations of the temporal mortality pattern. Seventh, while onset-to-door time, reperfusion strategy (emergency PCI vs. none), and binary use of mechanical circulatory support were available and reported, other reperfusion-related variables—including door-to-balloon time, post-PCI TIMI flow grade, procedural success, and precise timing of mechanical circulatory support relative to rupture—were not consistently documented in the electronic medical record across the full study period and could therefore not be analyzed. Prospective registries capturing granular procedural timing and success will be needed to clarify the contribution of reperfusion quality to rupture risk. Finally, several variables identified as associated with CR (e.g., Killip class III–IV, hypoalbuminemia, and delayed presentation) may partially reflect overall infarct severity rather than independent pathophysiological determinants; disentangling these pathways requires prospective, mechanistically oriented studies.

## 5 Conclusions

This study identified four variables independently associated with cardiac rupture after AMI: absence of emergency PCI, Killip class III–IV, female sex, and low serum albumin. The results also highlight important differences between rupture subtypes, with FWR showing substantially worse short-term survival than VSR. Surgical repair was associated with lower in-hospital mortality in selected patients. These findings may help inform closer monitoring and early multidisciplinary evaluation for high-risk AMI patients, but external validation is needed before routine clinical implementation.

## Data Availability

The original contributions presented in the study are included in the article/Supplementary Material, further inquiries can be directed to the corresponding author/s.

## Supplementary material

We received the Supplementary Material the manuscript. Please provide this as a separate Supplementary file.
