# Supplementary material for: Risk factors, subtype profiles, and outcomes of cardiac rupture after acute myocardial infarction: a case-control study
Source: Front Cardiovasc Med. 2026 Jun 10;13:1827832. doi: 10.3389/fcvm.2026.1827832 (PMC13290571; doi:10.3389/fcvm.2026.1827832)
Supplement: Supplementary file 1 [file Supplementaryfile1.docx]

**Supplementary Material**

*Risk factors, subtype profiles, and outcomes of cardiac rupture after acute myocardial infarction: a case-control study*

Dan Zhang, Chaojie Huang, Xiaosu Wang, Jingdan Yu, Litao Zhang*, Bo Liu*

**Supplementary Table S1.** Cox proportional hazards regression for in-hospital mortality within the cardiac rupture group.

| **Variable** | **HR** | **95% CI** | **P** | **PH test P*** |
| --- | --- | --- | --- | --- |
| FWR type | 3.22 | 1.69–6.14 | **<0.001** | 0.77 |
| Surgical repair | 0.19 | 0.07–0.51 | **0.001** | 0.49 |
| Killip III–IV | 0.51 | 0.29–0.91 | **0.023** | 0.29 |

*n = 70 (1 excluded due to missing length of stay); 56 events (deaths). Model fit using Breslow’s method for ties. HR = hazard ratio; CI = confidence interval. *Schoenfeld residual test for the proportional hazards assumption — all P > 0.05, indicating the assumption was not violated for any covariate. Bold values indicate statistical significance (P < 0.05).*

**Supplementary Table S2.** Firth-penalized logistic regression for in-hospital mortality within the cardiac rupture group.

| **Variable** | **OR (Firth)** | **Profile 95% CI** | **LR P** | **Standard logistic OR*** |
| --- | --- | --- | --- | --- |
| FWR type | 8.14 | 1.49–84.09 | **0.014** | 12.50 (1.30–119.97) |
| Surgical repair | 0.082 | 0.013–0.373 | **<0.001** | 0.060 (0.010–0.361) |
| Killip III–IV | 0.31 | 0.05–1.52 | 0.152 | 0.26 (0.04–1.60) |

*n = 71; 56 events. OR = odds ratio; CI = confidence interval based on profile penalized likelihood; LR P = likelihood-ratio-based p-value. *Standard (unpenalized) logistic regression results from Manuscript Table 6B provided for comparison. Bold values indicate statistical significance (P < 0.05).*

**Supplementary Table S3.** Sensitivity analysis of the early/late rupture cutoff.

| **Variable** | **≤2 vs >2 d (P)** | **≤3 vs >3 d [primary] (P)** | **≤4 vs >4 d (P)** |
| --- | --- | --- | --- |
| WBC (×10⁹/L) | 0.245 | **0.026** | **0.004** |
| Neutrophil count (×10⁹/L) | 0.124 | **0.031** | **0.003** |
| Albumin (g/L) | **0.003** | **0.023** | 0.071 |
| Admission cTnT (ng/L) | 0.078 | **0.015** | **0.040** |
| Onset-to-door (h) | **<0.001** | **<0.001** | **<0.001** |
| In-hospital death (%) | 0.840 | 0.400 | 1.000 |

*Group sizes: ≤2 vs >2 days (18 vs 53); ≤3 vs >3 days [primary] (28 vs 43); ≤4 vs >4 days (40 vs 31). Mann–Whitney U test for continuous variables; chi-square test for categorical variables. Bold values indicate statistical significance (P < 0.05).*

**Supplementary Figures**


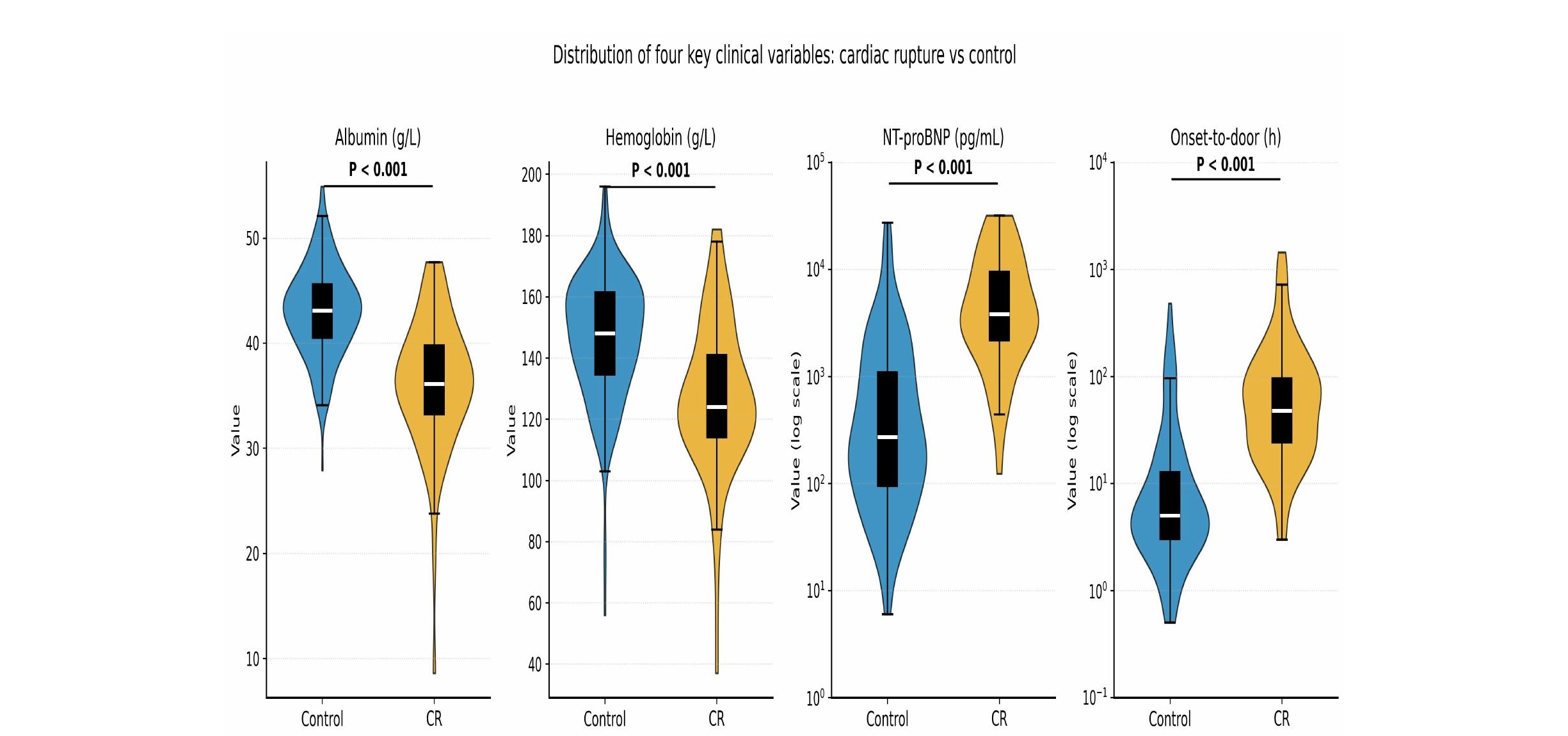


**Supplementary Figure 1. Distribution of four key clinical variables.** *Violin plots with box plots show median, IQR, and distribution shape. All four variables demonstrated highly significant differences between groups (all P<0.001). The CR group had lower albumin and hemoglobin, markedly elevated NT-proBNP, and substantially longer onset-to-door times. Sample sizes vary slightly across panels due to partial missing laboratory data, as indicated on the x-axis labels.*


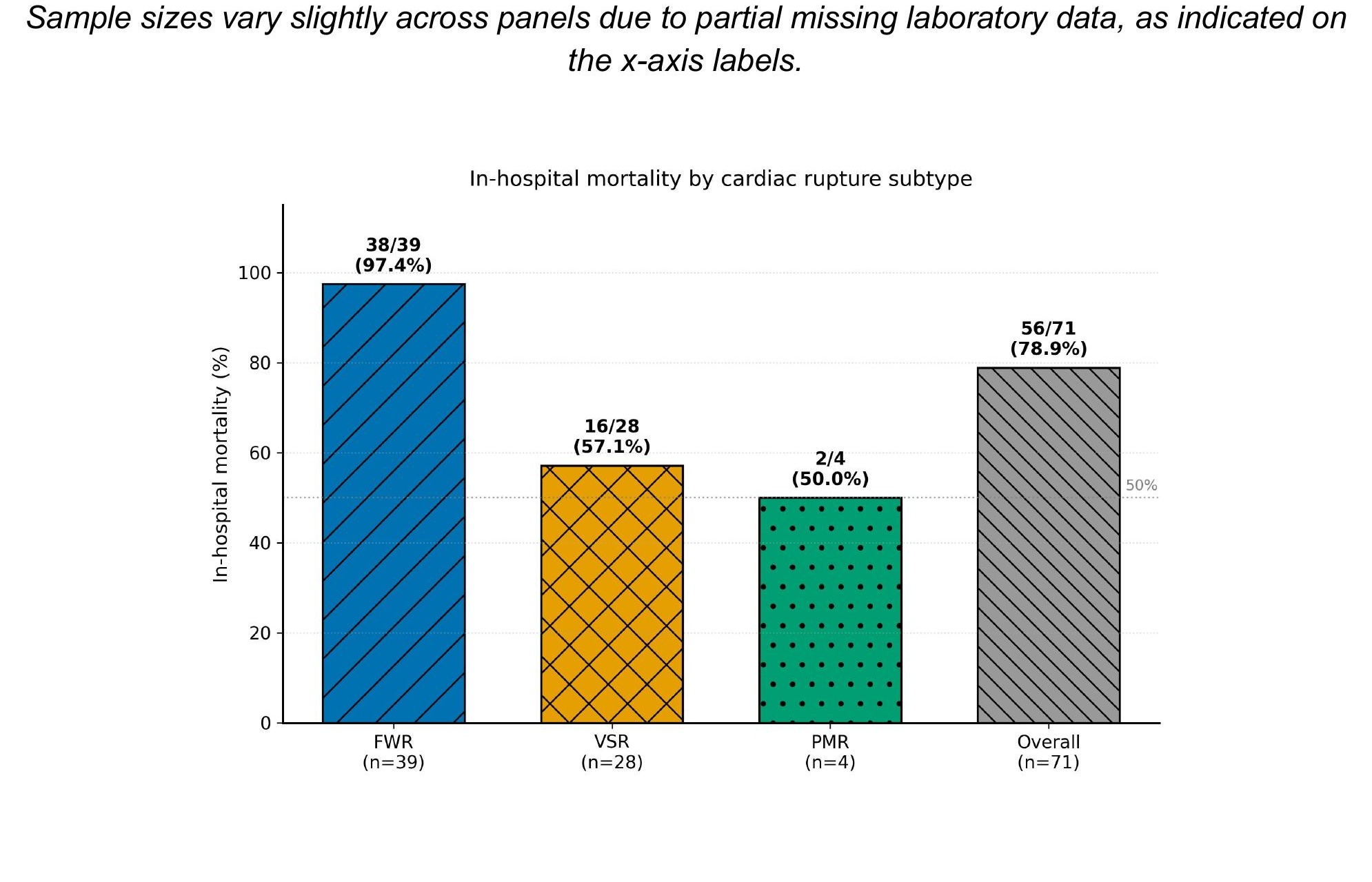


**Supplementary Figure 2. In-hospital mortality by cardiac rupture subtype.** *FWR carried near-universal mortality (97.4%, 38/39). VSR and PMR showed more favorable survival at 42.9% and 50.0% respectively, reflecting potentially treatable conditions with longer therapeutic windows. Overall cohort mortality was 78.9%.*
